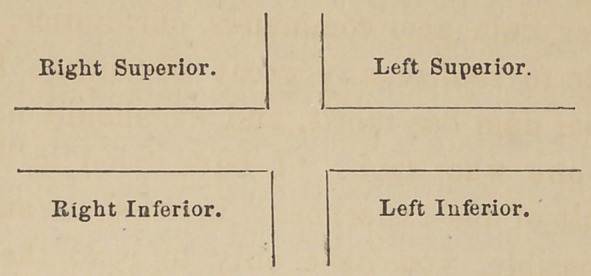# Forest City Society of Dental Surgeons

**Published:** 1870-07

**Authors:** Henri L. Ambler


### FOREST CITY SOCIETY OF DENTAL SURGEONS.

The Forest City Society of Dental Surgeons, held its annual meeting in Cleveland, Ohio; commencing June 7th, and continuing the greater part of two days. The following officers were elected for the ensuing year: President, C. R. Butler; Vice President, L. Buffett; Recording Secretary, H. L. Ambler; Corresponding Secretary, Chas. Buffett; Treasurer, B. Strickland; Councilmen, Corydon Palmer, A. Terry, F. S. Whitslar.

Dr. Corydon Palmer exhibited his improved paintings and models of the natural teeth. For utility and convenience in giving descriptions of teeth, or operations upon them; he separates the mouth into four divisions, thus:

| | |
|---|---|
| Right Superior. | Left Superior. |
| Right Inferior. | Left Inferior. |

Beginning and completing the circuit, viz: left superior, left inferior, right inferior, right superior. The teeth are to be numbered from one to eight, inclusive, beginning with the central incisor of each division, for number one. In speaking of a certain tooth he says such a number in such a division; for example, instead of saying right superior second bicuspid, it would be number five, right superior division, thus forming an intelligible and comprehensive system of practical benefit in discussions or elsewhere, bringing the Dentist at once to the point with few words.

In making a record of charges for operations upon the teeth, this system is brought into requisition, together with a regular set of characters to be used for specifying what kind of an operation had been made, and its position in the tooth. The practical working of this system was shown by the inventor as used by himself, having a number of strips of white paper ready ruled and arranged on a spring steel ring. He also exhibited some beautiful models. The first pair represented the teeth of a young Miss about fourteen years of age, showing the fissures and cavities, some of them having old fillings in. The second pair represented the same case as prepared for filling, all the cavities being excavated, and the teeth separated by locust wood. The third pair represented the case after all the operations had been completed with gold, following the best modern idea of contour operations, giving form and beauty to the teeth, by restoring their original cusps, depressions, and ridges; and in the fissures, cutting out those hair lines of decay which are so generally passed by, restoring the part removed, nicely with gold, for only in this way, can a thorough and permanent operation be made. The next pair represented the teeth of a young patient where the cuspids of the temporary set were still remaining, the superior, central, and lateral incisors being in an atrophied condition, having on their labial and lingual surfaces small pits and grooves, which ran lengthwise of the crowns, the cutting edges being notched.

This case was operated upon with gold, using a small finely serrated instrument, with an eight ounce mallet in his own hands, the filling when completed, mostly covering the labial and lingual surface, also the cutting edge. Then came his models of a case where a number of the superior and inferior teeth had been worn down. He restored a large portion of the crowns of cuspids, bicuspids, and molars; using gold, mallet, and coffer dam in all these operations, to attain *the highest.* He remarked that the length and size of shafts for instruments to be used in filling, should be in proportion to the form and delicacy of the point, all depending on what you desire to use the instrument for, and let each one be used in its proper place. Manufacturers are prone to make all the shafts of a size, or nearly so, not once considering their use, and the manner of point to be placed on certain ones. Vibration in shafts should be avoided as much as possible, for the gold can not be packed as well, and it is disagreeable to patients.

### SECOND DAY.

Drs. Slosson, Spelman and Ambler, were appointed a committee to investigate the cases near us, of those who were practicing Dentistry in violation of the law passed by this State. H. L. Ambler remarked that he had commenced to form some statistics especially in reference to the profession, hoping to derive interesting facts, to be set forth in the future.

Dr. Palmer remarked that the best operations of the present day, were made with adhesive gold foil. The non-adhesive was generally annealed before using, as an easier operation can be made with it, and it can be modeled to the walls of cavities quicker than the adhesive, but the operations when finished have not that good hard surface, which is so desirable for mastication and beauty, that will be found in fillings of adhesive foil, which, the more it is manipulated, the harder it becomes, which is one of its best qualities.

The President, Dr. C. R. Butler, assisted by H. L. Ambler, operated upon the following case: right superior cuspid, pulp alive, cavity on the posterior approximal surface, and of medium size; also, the right superior, first bicuspid, pulp alive, the anterior, posterior, and masticating surface being decayed. These were excavated so as to join, thus forming one continuous cavity, the posterior portion being very large, the teeth long, firmly set, and close together. The case was commenced by first applying coffer dam, from the first bicuspid on the left side, to the second molar on the right. Space was made by immediate wedging with locust wood, the excavating, filling, and finishing being done before removing the coffer dam. This occupied about three hours, thus completing a beautiful contour and operation.

H. L. Ambler reported the following case: "Mr. —, æt. 20, of nervo-bilious temperament, came to me with a swelling seated over the root of the left superior central incisor. About eight years ago as he was assisting to load a wagon when moving, something fell off and struck this tooth, breaking off the corner approximal to the other central; has never had any trouble with it, until within a few days; it has been aching considerably, but to-day the pain has gone, only leaving a soreness about the root. The day before I saw him, there was a discharge of pus through the left nares, the side on which the affected tooth was situated; also some through the mouth. Upon an examination, there could not be found upon the gums or palatine surface of the mouth, any point where pus had been evacuated, but evidently, it had burrowed its way into the inferior meatus, discharging anteriorly and posteriorly. I took a small drill and opened into the pulp chamber, from the lingual surface, found pulp decomposed, treated with carbolic acid, and filled." The above subjects were discussed by the different members, thus closing a pleasant session.

HENRI L. AMBLER, Rec. Sec.

## Figures and Tables

**Figure f1:**